# Regeneration of rabbit calvarial defects using cells-implanted nano-hydroxyapatite coated silk scaffolds

**DOI:** 10.1186/s40824-015-0027-1

**Published:** 2015-03-21

**Authors:** Jin-Young Park, Cheryl Yang, Im-Hee Jung, Hyun-Chang Lim, Jung-Seok Lee, Ui-Won Jung, Young-Kwon Seo, Jung-Keug Park, Seong-Ho Choi

**Affiliations:** Department of Periodontology, Research Institute of Periodontal Regeneration, Yonsei University College of Dentistry, Seoul, South Korea; Department of Medical Biotechnology, Dongguk University, Seoul, South Korea; Department of Dental Hygiene, College of Health Sciences, Eulji University, Seong-nam, Republic of Korea

**Keywords:** Bone regeneration, Silk scaffold, Nano-hydroxyapatite, Tissue engineering

## Abstract

**Background:**

The aim of this study was to characterize the efficacy of nano-hydroxyapatite-coated silk fibroin constructs as a scaffold for bone tissue engineering and to determine the osteogenic effect of human dental pulp and periodontal ligament derived cells at an early stage of healing in rabbits. 3D silk fibroin constructs were developed and coated using nano-hydroxyapatite crystals. Dental pulp and periodontal ligament cells from extracted human third molars were cultured and seeded onto the silk scaffolds prior to in vivo implantation into 8 male New Zealand White rabbits. Four circular windows 8 mm in diameter were created in the calvarium of each animal. The defects were randomly allocated to the groups; (1) silk scaffold with dental pulp cells (DPSS), (2) silk scaffold with PDL cells (PDLSS), (3) normal saline-soaked silk scaffold (SS), and (4) empty control. The animals were sacrificed 2 (n = 4) or 4 weeks (n = 4) postoperatively. The characteristics of the silk scaffolds before and after cell seeding were analyzed using SEM. Samples were collected for histologic and histomorphometic analysis. ANOVA was used for statistical analysis.

**Result:**

Histologic view of the experimental sites showed well-maintained structure of the silk scaffolds mostly unresorbed at 4 weeks. The SEM observations after cell-seeding revealed attachment of the cells onto silk fibroin with production of extracellular matrix. New bone formation was observed in the 4 week groups occurring from the periphery of the defects and the silk fibers were closely integrated with the new bone. There was no significant difference in the amount of bone formation between the SS group and the DPSS and PDLSS groups.

**Conclusion:**

Within the limitations of this study, silk scaffold is a biocompatible material with potential expediency as an osteoconductive scaffold in bone tissue engineering. However, there was no evidence to suggest that the addition of hDPCs and hPDLCs to the current rabbit calvarial defect model can produce an early effect in augmenting osteogenesis.

## Background

Bone grafting has numerous applications in dentistry such as alveolar ridge augmentation during implant therapy and reconstructive surgeries involving large bone defects. The preferred treatment is autograft, known to produce the best clinical outcome due to its osteogenic cellular component [1]; however, the use of autologous bone has its limitations due to donor site morbidity, increased surgery time and limited quantity [2,3]. Hence bone substitute materials of various organic and synthetic origins are commercially manufactured and used to provide the osteoconductive bulk upon which healing at the cellular level can be induced. Tissue engineering concepts have recognized the potential for graft materials to benefit from the addition of stem/progenitor cells aimed at enhancing the rate and quality of defect repair. According to the principles of tissue engineering, elements of successful bone regeneration include osteoblastic progenitor cells, suitable growth factors and an osteoconductive scaffold that allows the formation of vascular network [4,5].

The ideal requirements of bone tissue engineering scaffolds include biocompatibility, appropriate chemistry, morphology and structure to promote growth and differentiation of osteoblastic progenitor cells for synthesis of new bone matrix [5]. Silk fibroin is a promising biomaterial that has been used for both *in vitro* and *in vivo* applications due to the mechanical strength, biocompatibility, and a slow degradation rate that enables gradual replacement of fibroin with newly formed tissue [2]. Natural bone extracellular matrix consists of a loose network of collagen fibrils and hydroxyapatite crystals. Osteoconductive and osteoinductive properties of the scaffold can be reinforced by the addition of mineral hydroxyapatite (HA) due to its structural similarities to natural bone extracellular matrix. Incorporation of HA was shown not only to enhance the surface characteristics of the scaffold by increasing its roughness, but also promote viability of stem cells and their proliferation [6].

Cytocompatibility of silk scaffold and the subsequent formation of extracellular matrix onto silk fibroin have been demonstrated previously [7]. Addition of a cellular component may improve the osteoinductivity of a scaffold and help to increase the speed of bone formation. Various stem cells such as bone marrow stem cells, adipose tissue-derived stem cells and embryonic stem cells have been widely used in studies of bone tissue engineering [8-10]. Bone marrow derived mesenchymal stem cells (BMMSCs) in particular are the most studied, and have been shown to be capable of differentiating into multiple cell lineages including osteogenic, chondrogenic, adipogenic, myelosupportive stroma, myogenic, and neurogenic lineages. But due to certain shortcomings of obtaining the BMMSCs including pain, morbidity, and low cell number upon harvest, alternative sources for MSCs have been sought. Dental tissues are specialized tissues that do not undergo continuous remodeling; therefore dental tissue derived progenitor cells may be more restricted in differentiation potency in comparison to BMMSCs. Nevertheless, dental tissue derived cells can be easily acquired from tooth extractions and recent studies have proven dental stem cells to be a potent source for fabrication of bone structures [11]. This is due to their good osteogenic activity potentially as a result of their biological function to form hard mineralized tissue during tooth development [11]. Dental pulp stem cells (DPSCs) isolated from the dental pulp tissue could differentiate into osteoblastic precursors under stimulatory conditions, and were able to synthesize living fibrous bone tissue *in vitro*. Furthermore, following transplantation of the bone matrix into immunocompromised rats, lamellar bone tissues containing osteocytes were generated [12]. Several studies have demonstrated successful use of DPSCs seeded in scaffolds. For example, porous calcium phosphate ceramic discs [13], resorbable collagen sponges [14,15] and poly(lactic-co-glycolic acid) copolymers [16] have all been used to construct adequate cell-scaffold matrices for *in vivo* transplantations.

Osteogenic differentiation of cultured periodontal ligament stem cells (PDLSCs) has also been reported [17-19]. *In vivo* transplantation of PDLSCs into periodontal defects showed regeneration of the periodontal tissues, identified to be closely related to the adjacent trabecular bone, suggesting their involvement in alveolar bone regeneration [17]. Subsequent studies containing *in vitro* and *in vivo* experiments have shown use of PDLSCs with bone tissue engineering scaffolds such as nano-hydroxyapatite coated chitosan [20] and *in vitro*-derived extracellular matrix [21].

In the present study, a collective population of cells from the human dental pulp (DPCs) and periodontal ligament (PDLCs) were isolated and cultured. The rabbit calvarial defect model was used to evaluate the efficacy of the SF construct for bone regeneration *in vivo*, and to determine whether the addition of cellular component may improve the rate and quality of defect repair.

## Methods

### Preparation of silk scaffolds coated with nano-hydroxyapatite

Silk sutures were purchased from Won Corporation (Seoul, South Korea) and silk scaffolds were constructed using a weaving machine. The silk scaffolds were processed by extracting sericin, a glue-like protein that coats the native silk fibroin, using an aqueous solution containing 0.02 M Na_2_CO_3_ and 0.3% Ivory detergent. 0.15 g of nano-hydroxyapatite (Sigma) was dissolved in 10 ml of PBS and 1ml of the solution was dried on the silk scaffold (0.8×1.2 cm) in air. The scaffolds were then soaked in a 1% type atelocollagen solution (Bioland, Korea) and lyophilized in a freeze dryer (Samwon Freezing Engineering Co., Korea) at −80°C for 48 h. The silk scaffolds were incubated in 20 ml of 40% (v/v) ethanol containing 50 mM 2-morpholineoehtane sulfonic acid (MES, Fluka Chemic AG) (pH 5.5) for 30 min at room temperature. The silk scaffolds were then immersed in 20 ml of 40% (v/v) ethanol containing 50 mM MES (pH 5.5), 24 mM 1-ethyl-3-(3-dimethyl aminopropyl) carbodiimide (Fluka Chemic AG) and 5 mM N-hydroxysuccinimide (Fluka Chemic AG) for 12 h at room temperature. Once the reaction was complete, the composite silk scaffold was washed twice in 0.1 M Na_2_HPO_4_ (pH9.0) for 12 h. The scaffolds were washed twice in 1 M NaCl for 6 h, then in 2 M NaCl for 2 days and finally rinsed with distilled water. The washed scaffolds were lyophilized again and sterilized with γ-irradiation at 15 KGy.

### Primary cell culture

#### DPCs

Extracted third molars were used in experiments with the approval of patients. The patients provided informed consent for their tissue being used in accordance with guidelines approved by the Institutional Review Board, College of Dentistry, Yonsei University (IRB No. 2-2010-0009). The extracted third molars were washed with PBS containing antibiotic antimycotic (AA) (Welgene, Korea) for 3 mins after washing with 70% ethanol. The third molars were wavered with pliers and the dental pulp was removed. The pulp tissues were digested in a solution of 3 mg/mL collagenase type I (Sigma St. Louis, Mo., USA) and 4 mg/mL dispase (Siga, St. Louis, MO., USA) for 12 h at 4°C. The pulp tissue was then incubated in 3 mL of 0.25% trypsin for 15 min at 37°C. After trypsin digestion, the trypsin solution was diluted in alpha Modified Eagle’s Medium (α-MEM, Sigma) containing 10% fetal bovine serum (FBS, BioWhittakerTM, Cambrex, MD, USA). The cells were isolated from the dental pulp by pipetting. After centrifugation at 800 rpm for 5 min, the supernatant was removed and added to 10 mL of α-MEM. The cell suspensions were seeded in 100 mm culture dishes.

#### PDLCs

Human periodontal tissue was obtained from several extracted third molars of patients who had given their informed consent (IRB No. 2-2010-0009). The extracted third molars were washed with PBS containing antibiotic antimycotic solution (AA) (Welgene, Korea) for 3 min after washing with 70% ethanol.

The periodontal tissue was removed from the roots of the teeth and then divided into small pieces with scissors and tissues were digested in a solution of 3 mg/mL collagenase type I (Sigma, St. Louis, MO, USA) and 4 mg/mL Protease (Sigma, P 3417, USA) for 12 h at 4°C. Then, PDL tissues were incubated in 3 mL of 0.25% trypsin for 15 min at 37°C. After trypsin digestion, the trypsin solution was diluted with alpha Modified Eagle’s Medium (α-MEM, Sigma) containing 10% fetal bovine serum (FBS, BioWhittakerTM, Cambrex, MD, USA).

After centrifuging at 800 rpm for 5 min, the supernatant was removed and pipetted with 10 mL of α-MEM. The cell suspensions were seeded in 100 mm culture dish. The PDL fibroblasts from third to five passages were used.

### Seeding of cells in silk scaffolds

DPCs and PDLCs, suspended in alpha Modified eagle’s Medium (α-MEM, Sigma) supplemented with 10% fetal bovine serum (FBS, BioWhitekerTM, Cambrex Bioscience Walkersville, Inc., MD), were seeded onto the silk scaffolds. To obtain a very high cell seeding density, the cells were seeded onto the silk scaffolds under a dried condition. 100 μl of a harvested suspension – containing 2.8 × 10^4^ human dental pulp cells or 1 × 10^6^ human PDL cells – and silk scaffolds were placed in the petri dish in a humidified 5% CO_2_ incubator, and 3h later, 10 ml of α-MEM was added to the petri dish. The medium was replaced every 3 days for 7 days. After incubation for additional 7 days, the scaffolds were cultured in differentiation medium, which was replaced every 2 days for 3 weeks. The differentiation medium for DPCs was alpha Modified Eagle’s Medium (α-MEM, Sigma) supplemented with 10% fetal bovine serum (FBS, BioWhittakerTM, Cambrex, MD, USA), 1% AA, 100 nM dexamethasone, 0.05 mM Ascorbic acid and 10 mM β-glycerophospate. The differentiation medium for PDLCs was α-MEM containing 10% FBS, 10 mM β-glycerophosphate (Sigma), 50 μM L-ascorbate2-phosphate (Sigma) and 10^−7^ M dexamethasone (Sigma). The result of cell-seeding procedure was checked with scanning electron microscopy.

### Animals

Eight male New Zealand White rabbits weighing 2.8-3.2 kg and age 16–20 weeks were used in this study. Animal selection and management, surgical protocol and preparation followed the routines approved by the Institutional Animal Care and Use Committee, Yonsei Medical Center, Seoul, Korea.

### Study design

Four circular defects (8mm diameter) were created in each animal, which were randomly allocated to the four study groups including PDLC-silk scaffold group (PDLSS group), DPC-silk scaffold group (DPSS group), normal saline-soaked silk scaffold group (SS group) and an empty control group. The animals were euthanized at either 2 or 4 weeks postsurgery.

### Surgical protocol

**Figure 1:**
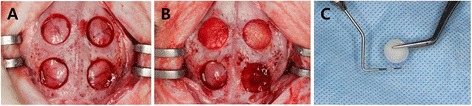
**Surgical procedure. A**. Four standardized defects 8 mm in diameter were created using a trephine bur. **B**. The defects in each animal were assigned into four groups. Clockwise from bottom left: sham control, silk scaffold (SS), dental pulp cells seeded on SS (DPSS), periodontal ligament cells seeded on SS (PDLSS). **C**. The silk scaffold was made at 8 mm in diameter in order to fit into the defects.

General anaesthesia was induced in all animals using intramuscular injections of zoletil (15 mg/kg) and rompun (5 mg/kg). The head of the rabbit was shaved and disinfected using povidone iodine prior to local anaesthetic injections at the surgical site using 2.2 ml Lidocaine Hydrochloride 2% with adrenaline 1:80,000. An incision was made along the midline from the frontal bone to the occipital bone and a full-thickness flap was elevated. Under copious saline irrigation, four standardized round defects 8 mm in diameter were created using a trephine bur (Figure 1). The resected bones were removed carefully to avoid injury to the underlying brain tissue. The defects were filled with the experimental materials according to the assigned group. The flaps were repositioned and sutured with resorbable suture material.

The animals were sacrificed at either 2 or 4 weeks postsurgery. The skin flaps were reflected to expose the periosteum and the entire calvarium was resected and harvested from each animal.

### Histologic processing

Block sections of the surgical sites were fixed in 10% formalin for 10 days. The fixed specimens were decalcified in 5% formic acid for 14 days and then embedded in paraffin. Serial 5 μm thick sections were cut along the midline of the calvarial defects. Only sections located at the middle of the defects were selected, and stained with hematoxylin-eosin for histologic and histometric analysis.

### Analysis methods

#### Histomorphometric analysis

After the conventional microscopic examination, computer-assisted histometric measurements in the calvarial defect were performed using an automated image analysis system (Image-Pro Plus; Media Cybernetics, Silver Spring, MD). The following parameters were measured from each histologic section of the defect areas.

- Total augmented area (mm^2^) – Total sum of the area of new bone, residual particles, connective tissue, adipose tissue, blood vessels within the defect area.
- Residual material (mm^2^) – Area of the remaining silk scaffold within the defect.
- New bone (mm^2^) – Area of newly formed bone within the defect.

### Statistical analysis

The statistical analysis was performed using a commercially available software program (SPSS 18.0, SPSS, Chicago, IL). Histomorphometric records from the calvarial defect samples were used to calculate the mean and standard deviation (SD) values of groups. One-way analysis of variance (ANOVA) was used to analyze the difference between the groups at 2 and 4 weeks. Statistical significance was considered when P <0.05.

### SEM observations

The morphology of nHA-coated scaffolds before and after the cell-seeding procedure were observed by scanning electron microscopy (SEM; S-3000N, Hitachi, Tokyo, Japan) at an accelerating voltage of 30 kV.

### Clinical and histological findings

Throughout the healing periods, the animals were carefully monitored for adverse reactions around the surgical site. The specimens were examined by a single, blinded examiner with the aid of a binocular microscope (DM LB, Leica Microsystems, Wetzlar, Germany) equipped with a camera (DC300F, Leica Microsystems). Images of the slides were acquired and saved as digital files.

## Results

### SEM observations of nHA-coated silk scaffolds

**Figure 2:**
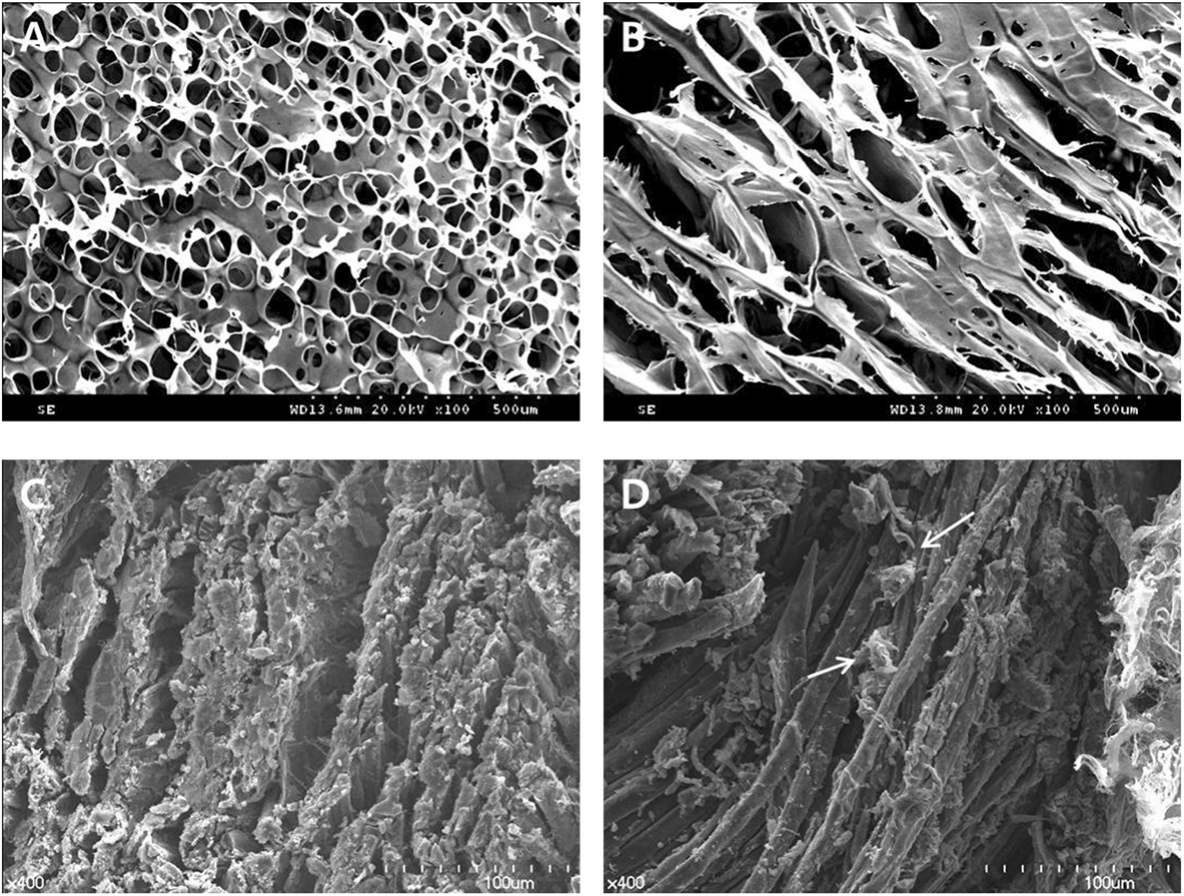
**Scanning electron microscopy images. A** and **B** shows the cross section and longitudinal section of the silk scaffold respectively prior to the cell culture. Porous, interconnected structure can be observed, ×100 magnification. **C** and **D** show the cross section and longitudinal section of the silk scaffold respectively, following the cell culture, ×400 magnification. Arrows represent spreading of the cells with secreted extracellular matrix on the silk fibroin. Arrowheads represent nHA crystals on silk fibroin.

SEM observations of the surface of the nHA-coated silk scaffold revealed a combination of porous architecture of the silk fibroin. A closer examination at a higher magnification revealed organization of the fibrous structures into bundles. Cross-sectional view revealed interconnected porous structure of the silk fibroin construct. Pore sizes varied between 20–80 μm with mean pore diameter of 65 μm (Figure 2A). Longitudinal sections showed an internal structure with fibrous bundles mainly oriented parallel to the scaffold surface.

At 400 magnification view (Figure 2C, D) homogenous distribution of nano-HA crystals on pure silk fiber substrates can be observed as small round granules. In parts, nHA crystals displayed formation of clusters with attachment of the cellular matrix. The implanted cells were well spread out and attached to the surfaces of scaffolds. Also, the formation of an ECM was observed. This is a typical response that is indicative of cytocompatibility and adaptation of the cells onto silk fibroin.

### Clinical findings

Healing of all surgery sites was without any adverse reactions or postoperative complications such as abnormal bleeding or infection. Signs of inflammation such as swelling appeared to be minimal, and the grafted materials were confirmed to be intact within the defects at the time of sacrifice and sample collection.

### Histologic & histomorphometric analysis

In the control group, negligible amount of new bone formation was observed. The defect spaces were filled by the underlying brain tissues as well as the overlying periosteum that was collapsed into the vacant space.

In DPSS, PDLSS and SS groups, the silk fibroin constructs occupied the defect spaces and exhibited well-maintained structure in all sites. The scaffolds were superficially covered by the overlying periosteum and the silk fibers were surrounded by the connective tissue of ECM comprising loose collagen network stained by the light blue coloration of Masson’s trichrome staining.

**Figure 3:**
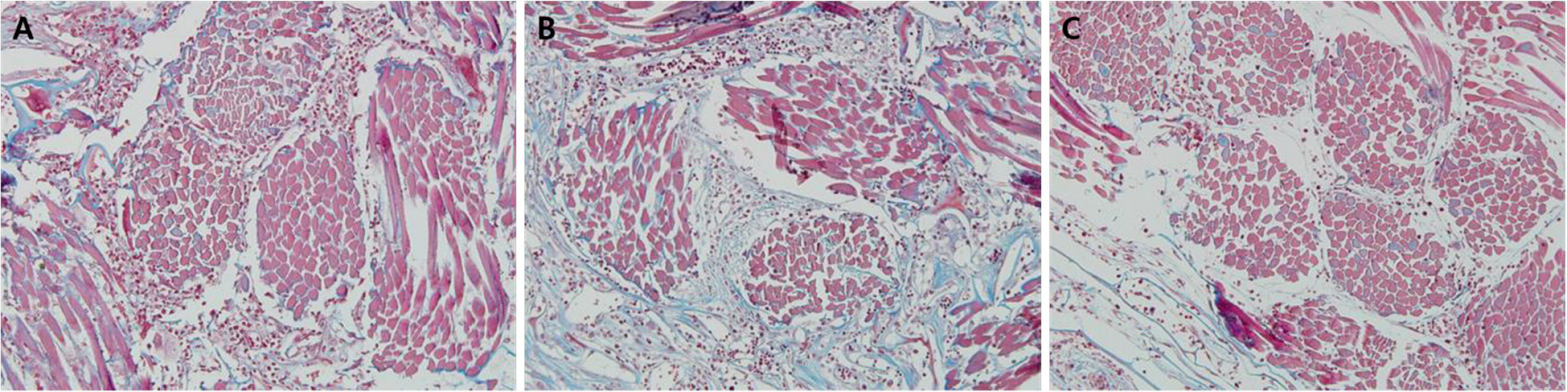
**Histological view of the groups at 2 weeks postoperatively. A** – DPSS group, **B** – PDLSS group, **C** – SS group. All experimental groups showing inflammatory cells mainly macrophages evenly distributed around the silk scaffold. ×200 magnification, Mason’s trichrome staining.

**Figure 4:**
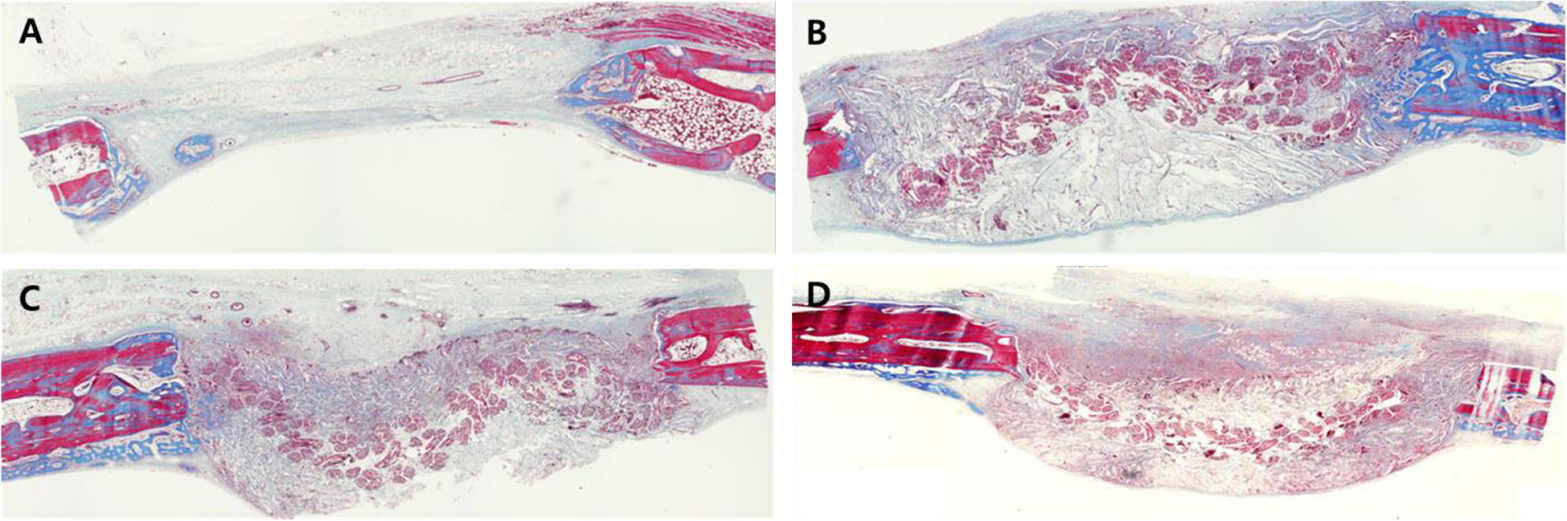
**Histological view of the groups at 2 weeks postoperatively. A** –control group shows negligible amount of bone formation. Minimal bone formation observed in: **B** – SS group, **C** – PDLSS group, **D** – DPSS group, ×40 magnification, Mason’s trichrome staining.

At 2 weeks, the interconnected porous structures were occupied by the inflammatory cells mainly macrophages, which were evenly distributed (Figure 3). The scaffold showed compact arrangement of silk fibers organized into bundles and blood vessels formed vascular networks around the bundles of silk fibroin. Minimal new bone formation was observed (Figure 4).

**Figure 5:**
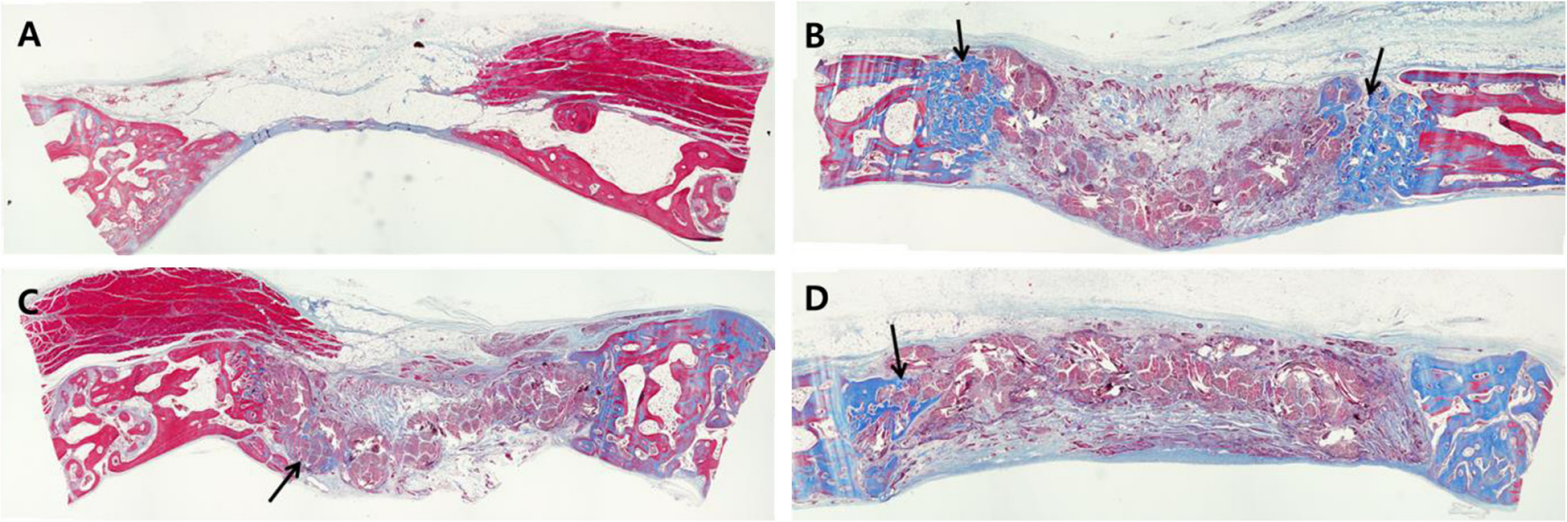
**Histological view of the groups at 4 weeks postoperatively. A** – Control group shows negligible amount of bone formation. **B** – SS group, **C** – PDLSS group, **D** – DPSS groups all display early bone formation at the periphery of the defects shown by the arrows. ×40 magnification, Mason’s trichrome staining.

At 4 weeks, the inflammatory cells no longer existed around the fibrous network. Angiogenesis had occurred surrounding the fibrous bundles evident by the appearance of blood vessels, however none were seen to penetrate the internal structure of the porous network. ECM appeared visibly denser than at 2 weeks and early new bone formation was observed occurring mainly from the periphery of the defects. The newly formed bone was seen to be in close contact with the individual fibers of silk scaffold (Figure 5).

**Figure 6:**
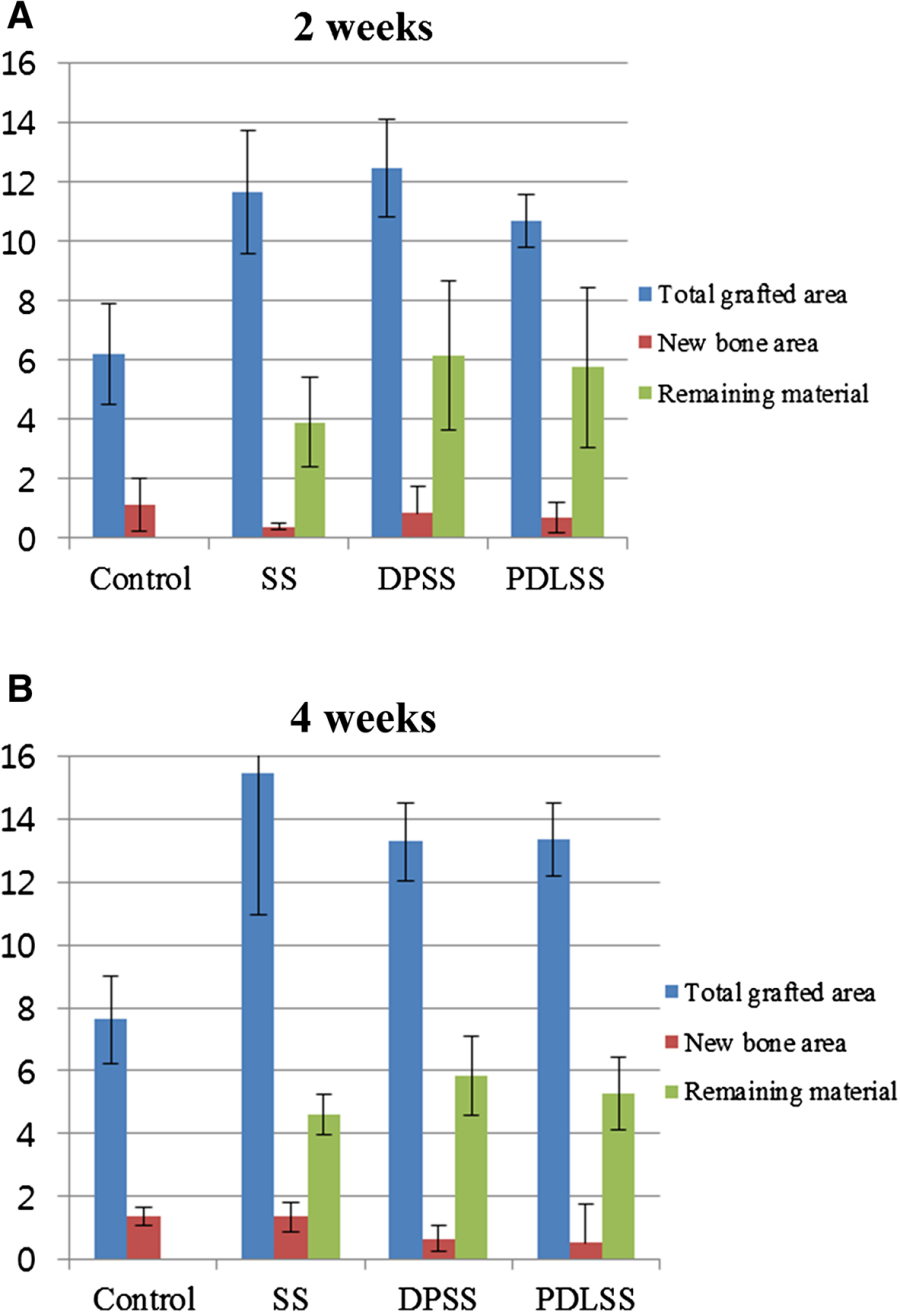
**Graphs showing total grafted area, new bone area, remaining material (mm** ^**2**^ **). A –** after 2 weeks of healing, **B** – after 4 weeks of healing.

Histomorphometric analysis revealed no statistical significance between DPSS, PDLSS and SS groups in terms of the area of new bone (Table 1, Figure 6). However, the total augmented area showed to be significantly greater in the DPSS, PDLSS and SS groups compared to the control group, which invariably is due to the unresorbed silk fiber constructs at both 2 and 4 weeks. There was no remarkable difference in the appearance of the remaining silk fibers between the two time periods in all groups, and histomorphometric analysis showed no significant difference in the area of residual material between 2 and 4 weeks.

**Table 1:** **Total grafted area, new bone area, remaining material at 2 and 4 weeks**

	**Parameters (mm** ^**2**^ **)**	**Control**	**SS**	**DPSS**	**PDLSS**
2 Weeks	Total grafted area	6.2 ± 1.7	*11.66 ± 2.1	*12.46 ± 1.63	*10.68 ± 0.91
	New bone area	1.13 ± 0.88	0.4 ± 0.13	0.84 ± 0.93	0.7 ± 0.5
	Remaining material	NA	3.9 ± 1.5	6.15 ± 2.5	5.77 ± 2.7
4 Weeks	Total grafted area	7.63 ± 1.38	*15.47 ± 4.37	*13.28 ± 1.23	*13.35 ± 1.17
	New bone area	1.37 ± 0.29	1.36 ± 0.47	0.67 ± 0.39	0.54 ± 1.23
	Remaining material	NA	4.61 ± 0.62	5.83 ± 1.27	5.29 ± 1.15

*Significantly different from the control (P < 0.05).

SS – silk scaffold.

DPSS – dental pulp cells with silk scaffold.

PDLSS – periodontal ligament cells with silk scaffold.

## Discussion

Silk fibroin is a promising material due to its excellent mechanical strength, biocompatibility and porous architecture to act as the artificial extracellular matrix required for 3D tissue engineering application. The objectives of this study were to evaluate the efficacy of silk fibroin constructs for the regeneration of bone defects and determine the effect of the added cellular components *in vivo*. The current experimental model has previously been shown to be suitable for the evaluation of bone substitutes at the early stage of healing when study periods of 2 and 4 weeks were combined [22]. Negligible amount of bone formation was noticed in the defects of the control group at both 2 and 4 weeks as the surrounding soft tissue structures appeared to have collapsed into the defect spaces. The histomorphometric analysis shows that all the experimental groups had greater total augmented area than the control group at both 2 and 4 weeks, however, no significant difference was revealed in regards to the area of new bone between the groups. This result indicates that the silk scaffold was effective in maintaining the defect space for at least 4 weeks, but the addition of cellular components had no effect in the amount of bone regeneration.

Silk fibroin scaffolds have been demonstrated to be mechanically robust in previous studies [23,24]. In the present study, the grafted volumes were well maintained in all of the experimental groups which had led to new bone formation occurring from the periphery of the defects. The early new bone appeared to be in close contact with the fibrous bundles of the silk scaffold, and the provisional collagen matrix seemed to surround the individual silk fibers without any adverse reaction. This histologic evidence demonstrates biocompatibility of the silk fibroin *in vivo* to act as the osteoconductive basis for bone regeneration. Silk fibroin is known for its biocompatibility and low immunogenicity following implantation once rendered devoid of sericin, a glycosylated protein emitting immunogenicity [25]. In vitro studies have demonstrated that silk is enzymatically broken down by a family of proteases directly into peptides and amino acids, which indicates that the biodegradation products of silk fibroin do less or no harm to the human body [26]. In this study, inflammatory cells mainly macrophages were found evenly distributed within the scaffold at 2 weeks. However, no abnormal clinical findings were observed during follow up of the animals, and the inflammatory cells had completely vanished at 4 weeks suggesting that this would have been part of the normal wound healing sequence. Macrophages are known to predominantly appear during the inflammatory phase of the wound healing and contribute to inducing angiogenesis and laying down a new ECM. At 4 weeks, denser collagenous provisional matrix was observed throughout the scaffold, evident by the increased blue coloration around the SF by the Masson’s trichrome staining and formation of blood vessels around the fibrous bundles. However, the vascular network failed to penetrate the bundles of silk fibers. This could be due to the compact organization of the fibrous bundles as well as the lack of biodegradation of the material in order to accommodate sufficient space for angiogenesis.

The silk fibroin constructs remained unresorbed after 4 weeks in this study. This is concurrent with previous literature in which silk was demonstrated to be proteolytically biodegraded and resorbed *in vivo* typically within a year [26]. The slow biodegradation of silk may be responsible for the small amount of bone formation in all experimental groups, as new bone formation cannot occur if the defect space is already occupied. *In vivo* degradation kinetics of the silk scaffold should closely match the functional needs and mechanical properties specific to the defect type [27]. In consideration of slow degradation, mechanical toughness and the pattern of bone formation occurring from the periphery, SF constructs could be advantageously used in highly loaded areas where cortical bridging and long-term support of function is critical. In non-stress bearing defects such as the one used for this study, long term support may not be necessary but rather dynamic resorption of the implant material and bone remodeling occurring synchronously in a gradual substitution process [28]. Hence, timely resorption of SS can be considered favorable in the promotion of osteogenesis. It has been shown that resorption rate of silk fibroin can be modulated by varying the pore size, crystallinity and molecular weight distribution [29]. Wang et al. have shown that the *in vivo* behavior of silk fibroin can be predictably altered and controlled to match the tissue-specific requirements of the scaffold characteristics and resorption rates [30]. Furthermore, acid-degraded silk fibroin fragments with low molecular weight has been shown to produce highly osteogenic properties by inducing increases in ALP, type-I collagen and fibronectin levels [31].

The present study incorporated the use of nHA-coated silk fibroin. Although a control group containing pure silk scaffold without nHA-coating may have demonstrated potential benefits of the nHA-coating, the primary end point in the current study was to investigate the effects of the addition of two cellular components in osteogenesis. Furthermore, in vitro studies [32,33] have demonstrated that nHA-coating on silk fibroin may improve cytocompatibility compared to the pure silk fibroin. Fibrous substrates have been shown to support cell attachment, proliferation and differentiation, while the hydroxyapatite constituent promotes the secretion of ECM for mineralization of new bone [34]. The degradation of nHA changes the Ca/P metabolism and triggers the activation of osteoblasts [35]. The nHA particles showed higher calcium release and increased osteoblast attachment than the conventional sized HA. The SEM appearance of the nHA crystals with overlying ECM is a concurrent finding to the reports in previous studies. In vitro study by Wei et al. [36] suggested that surface topography had a crucial influence on cell behavior, which was accompanied by attenuated proliferation rates on rougher surfaces. Nevertheless, after 2 weeks of cultivation, cell number of osteoblasts on mineralized silk was slightly higher than that on pure silk.

In the present study, the DPCs and PDLCs were cultured and seeded onto the nHA-coated SF constructs for 3 weeks. It has been shown that pre-culture of cells on silk scaffold prior to implantation improved the distribution of ECM mineralization due to better cell distribution within the scaffold [7]. Ideally, greater quantity of ECM should be produced with a larger number of cells. However, large cell numbers on silk scaffolds might be counter-productive for cell-attachment due to limited scaffold surface area. The cells were prepared according to a coherent protocol to existing literatures, in which osteoblastic precursors were obtained *in vitro*, and successful bone regeneration was achieved *in vivo* [11,12,20,37]. The SEM observations of the silk fibroin constructs after cell culture showed presence of the cellular components surrounded by ECM.

The silk scaffold used in the present study contained mean pore diameter of 65 μm. Due to scaffold shrinkage during drying this pore size is likely to be a reduced figure, which would be reversible when scaffolds were again placed in liquid. A previous study [7] demonstrated that small scaffold pores with average pore diameters below 100 μm did not constrain medium penetration into the middle of the scaffold, even in a static set-up. On the other hand, the smallest pores (61.6 ± 5.0 μm) presented the largest growth surface to the cells and allowed better migration of cells to the inner most parts of the scaffold resulting in more homogenous distribution of mineralized tissue, despite the general belief that larger pores improve cell and medium distribution.

However, histologically at 2 and 4 weeks *in vivo*, no significant difference in the amount of bone formation was observed in both DPSS and PDLSS groups compared to the SS group. A possible explanation could be that the proportion of stem cells within the cultured population may not have been enough to produce a significant impact on bone formation. The purity of the stem cells could have been enriched by initial validation of their ability to generate clonogenic cell clusters *in vitro* followed by further isolation of the stem cells using protocols such as magnetic bead cell separation based on the expression of mesenchymal stem cell associated markers [38].

In addition, xenogeneic cells of human origin were transplanted into rabbits that were not immunocompromised. The lack of host-verses-graft response at the grafted sites may be attributed to the initial scarcity of blood vessels at the site of implantation as well as the isolated nature of the contained-type defects in the calvaria. Had allogeneic cells been used, it remains possible that greater synergy might have been achieved. Previous studies have shown that although xenogeneic supply of mesenchymal stem cell (MSC) are likely to be rejected by the host after transplantation, allogeneic MSCs are well-tolerated by the recipient hosts [39]. DPSCs have also been shown to possess immunosuppressive effects [40].

Furthermore, it is impossible to verify in the present study whether new bone originated from the grafted cells or native cells. Future studies should employ cell labelling techniques to confirm that neo-osteogenesis derived from the recruited hDPCs and hPDLCs rather than the endogenous rabbit MSCs that could have migrated to the defect site.

## Conclusions

In summary, the results supported the biocompatibility of nHA-coated silk and its potential usefulness as an osteoconductive scaffold. However, there is a lack of evidence to suggest that using the current method of preparation, hPDLCs and hDPCs can produce an early effect in bone regeneration. Resorption rate of the silk fibroin should be modulated in order to promote timely substitution of the silk fibroin by living tissues. Further studies should be performed using confirmed stem cell population with longer healing periods for proper evaluation of its effects.

### Availability of supporting data

The data sets supporting the results of this article are included in the article.
